# Comparison of Qualitative and Quantitative Analyses of MR-Arterial Spin Labeling Perfusion Data for the Assessment of Pediatric Patients with Focal Epilepsies

**DOI:** 10.3390/diagnostics12040811

**Published:** 2022-03-25

**Authors:** Domenico Tortora, Matteo Cataldi, Mariasavina Severino, Alessandro Consales, Mattia Pacetti, Costanza Parodi, Fiammetta Sertorio, Antonia Ramaglia, Erica Cognolato, Giulia Nobile, Margherita Mancardi, Giulia Prato, Laura Siri, Thea Giacomini, Pasquale Striano, Dario Arnaldi, Gianluca Piatelli, Andrea Rossi, Lino Nobili

**Affiliations:** 1 Neuroradiology Unit, IRCCS Istituto Giannina Gaslini, 16147 Genoa, Italy; costanzaparodi@gaslini.org (C.P.); fiammettasertorio@gaslini.org (F.S.); antoniaramaglia@gaslini.org (A.R.); andrearossi@gaslini.org (A.R.); 2 Child Neuropsychiatry Unit, IRCCS Istituto Giannina Gaslini, 16147 Genoa, Italy; matteocataldi@gaslini.org (M.C.); ericacognolato@gaslini.org (E.C.); giulianobile@gaslini.org (G.N.); margheritamancardi@gaslini.org (M.M.); giuliaprato@gaslini.org (G.P.); laurasiri@gaslini.org (L.S.); theagiacomini@gaslini.org (T.G.); linonobili@gaslini.org (L.N.); 3 Department of Neuroscience, Rehabilitation, Ophthalmology, Genetics, Child and Maternal Health (DINOGMI), University of Genoa, 16147 Genoa, Italy; pasqualestriano@gaslini.org (P.S.); dario.arnaldi@gmail.com (D.A.); 4 Division of Neurosurgery, IRCCS Istituto Giannina Gaslini, 16147 Genoa, Italy; alessandroconsales@gaslini.org (A.C.); mattiapacetti@gaslini.org (M.P.); gianlucapiatelli@gaslini.org (G.P.); 5 Pediatric Neurology and Muscular Diseases Unit, IRCCS Istituto Giannina Gaslini, 16147 Genoa, Italy; 6 IRCCS Ospedale Policlinico San Martino, 16147 Genoa, Italy; 7 Department of Health Sciences DISSAL, University of Genoa, 16147 Genoa, Italy

**Keywords:** focal epilepsy, arterial spin labeling, magnetic resonance, perfusion, seizure onset zone

## Abstract

The role of MR Arterial-Spin-Labeling Cerebral Blood Flow maps (ASL-CBF) in the assessment of pediatric focal epilepsy is still debated. We aim to compare the Seizure Onset Zone (SOZ) detection rate of three methods of evaluation of ASL-CBF: 1) qualitative visual (qCBF), 2) z-score voxel-based quantitative analysis of index of asymmetry (AI-CBF), and 3) z-score voxel-based cluster analysis of the quantitative difference of patient’s CBF from the normative data of an age-matched healthy population (cCBF). Interictal ASL-CBF were acquired in 65 pediatric patients with focal epilepsy: 26 with focal brain lesions and 39 with a normal MRI. All hypoperfusion areas visible in at least 3 contiguous images of qCBF analysis were identified. In the quantitative evaluations, clusters with a significant z-score AI-CBF ≤ −1.64 and areas with a z-score cCBF ≤ −1.64 were considered potentially related to the SOZ. These areas were compared with the SOZ defined by the anatomo-electro-clinical data. In patients with a positive MRI, SOZ was correctly identified in 27% of patients using qCBF, 73% using AI-CBF, and 77% using cCBF. In negative MRI patients, SOZ was identified in 18% of patients using qCBF, in 46% using AI-CBF, and in 64% using cCBF (*p* < 0.001). Quantitative analyses of ASL-CBF maps increase the detection rate of SOZ compared to the qualitative method, principally in negative MRI patients.

## 1. Introduction

Worldwide, it is estimated that 10.5 million children under 15 years have active epilepsy, representing about 25% of the global epilepsy population [1]. Approximately 60% of epilepsy patients suffer from focal seizures, and in approximately 15% of these patients, seizures are not adequately controlled with anticonvulsive drugs, and such patients are potential candidates for surgical treatment [2]. Surgical treatment may provide an effective curative solution for patients with focal epilepsy when it leads to the removal of the epileptogenic zone (EZ), the area of the cortex that is necessary and sufficient for initiating seizures and whose removal (or disconnection) is necessary for complete abolition of seizures [3]. Thus, a comprehensive pre-surgical evaluation with precise delineation of the EZ is crucial for an optimal outcome. Indeed, analysis of seizure semiology, interictal, and ictal electrophysiological recordings, and structural MRI are considered first-line evaluations in EZ localization [4]. Nevertheless, when first-line non-invasive evaluation yields inconsistent conclusions or structural MRI is uninformative, other studies become necessary. Interestingly, a recent meta-analysis demonstrated a significantly higher proportion of MR-negative cases in children compared to adults [5]. Although in these cases stereo-electroencephalography (SEEG) is still considered the “gold standard” diagnostic procedure for localizing the EZ, additional information from different modalities is required before performing any invasive procedure [4,6]. Nevertheless, further assessments with non-invasive functional neuroimaging techniques (NIFNTs) may be a practical option to guide or even avoid SEEG in selected cases [7].

Among NIFNTs, advanced nuclear imaging techniques are established methods used to assess metabolic changes associated with the EZ [6]. In particular, interictal PET and ictal SPECT are widely used to identify the EZ in the pediatric population, showing an accuracy of 60−80% (interictal PET) [8] and 70–82% (ictal SPECT) [9]. These imaging techniques are relatively invasive for children, as they require exposure to radiation, as well as intravenous injections of radioactive tracers and/or contrast agents. Therefore, it would be favorable to find less invasive approaches for localizing the EZ in children.

In the last few years, improvements in imaging acquisition techniques have been made to reduce the invasiveness of studies that evaluate brain perfusion and metabolism, especially in children. In particular, Arterial Spin Labeling (ASL) represents a novel MR perfusion technique enabling direct, non-invasive measurements of CBF without the need for contrast material injection or exposure to ionizing radiation. Indeed, it employs magnetically labeled blood-water protons as an endogenous diffusible tracer to noninvasively estimate whole-brain perfusion. Briefly, radiofrequency pulses are used to invert the magnetization of blood water protons at the level of major arteries of the neck. Then, after a delay to allow for labeled molecules to flow into the brain tissue, “labeled” MR brain images are acquired that contain signals from both labeled water and static tissue water. Subsequently, separate “control” images are acquired without prior labeling of arterial protons, and the signal difference between “control” and “labeled” images provides a measure of labeled blood from arteries delivered to the brain, which can be used to quantify CBF [10]. Nagesh et al. recently demonstrated the complementary role of ASL MR perfusion in the localization of EZ, based on CBF changes related to seizure activity [11]. Despite the potential benefits of the ASL MRI technique related to the lack of contrast material and radiation requirements, studies performed on patients with epilepsy report inconsistent findings regarding the perfusion changes observed during and after seizures. Moreover, the ASL literature on pediatric patients remains inconclusive, particularly regarding the best approach needed to interpret CBF maps in patients with epilepsy. Of note, several approaches have been proposed to localize the EZ on CBF maps, including standard qualitative visual analysis [12,13] and more complex quantitative approaches evaluating the asymmetry of CBF at a voxel level [14,15].

We hypothesized that quantitative voxel-based analysis of CBF may increase the accuracy of the seizure onset zone (SOZ; area of cortex that initiates clinical seizures) [3] detection compared to the qualitative visual approach, which is widely used in clinical routine. Thus, we prospectively assessed the detection rate of ASL perfusion in the localization of the SOZ in two groups of children with MRI-positive and MRI-negative focal epilepsy, comparing three different methods of CBF analysis: (i) the qualitative visual analysis (qCBF); (ii) the quantitative voxel-based index of asymmetry (AI-CBF); and iii) a novel quantitative approach that compares at voxel-level CBF of each patient with baseline normative ASL data sets of an age-matched healthy population (cCBF).

The main aim of the study was to compare the SOZ detection rate of qualitative versus quantitative ASL analyses. Therefore, we included pediatric patients with focal epilepsy without considering etiology or drug response to increase the number of subjects and strength of statistics of results. Moreover, the study did not aim to evaluate the usefulness and added value of ASL in presurgical assessment for the identification of EZ, which will be done in a further study in a selected cohort of subjects all undergoing epilepsy surgery. For the same reason, we decided to consider the concept of “Seizure Onset Zone”, avoiding the term “Epileptogenic Zone”, since our purpose was not strictly related to epilepsy surgery.

## 2. Methods

### 2.1. Patients

Children with focal epilepsy consecutively referred to our institute from 1 January 2017 to 30 June 2020 were included. All patients underwent an anatomo-electro-clinical assessment, including discussion of ictal semiology, video-EEG, 3T brain MRI, and FDG-PET and Electrical Source Imaging in some cases. Inclusion criteria were: (i) confirmed unilateral focal epilepsy and (ii) presence of an MR perfusion study with the background-suppressed 3D-PCASL technique. Exclusion criteria were: (i) motion artifacts on MR images and (ii) undefined epilepsy. According to the imaging findings, included subjects were stratified into two groups: (i) positive-MRI when a brain lesion related to epilepsy was found and (ii) normal-MRI when no lesions were found in the brain MRI. In a restricted group of subjects undergoing epilepsy surgery, the seizure outcome was clinically followed after surgery. Outcome data at one year postoperative were based on Engel’s classification [16].

We also retrospectively evaluated 3D-PCASL studies of a group of healthy pediatric patients who performed 3T brain MRI examinations between 2016 and 2020 for minor trauma or headache. Inclusion criteria for this group were (i) normal brain MRI, (ii) normal MR-angiography, and (iii) normal 3D-pCASL perfusion, defined according to the absence of inter-hemispheric CBF asymmetry at both qualitative and quantitative evaluations.

Of note, in this study we addressed the issue related to the different trajectories of CBF development during childhood, dividing all subjects into two separate age-groups: (i) ≤7 years of age, typically showing higher mean CBF, and (ii) >7 years of age, normally showing a trend of CBF reduction [17]. Comparisons between subjects with epilepsy and controls were performed within each age group.

### 2.2. MR Imaging

MRI studies were performed for both patients and controls with a 3T scanner using a 32-channel head array coil (Ingenia Cx, Philips, Best, the Netherlands). The MRI examinations included conventional sequences, such as 3D-T1-weighted, T2-weighted, and susceptibility-weighted (SWI) images, and pCASL perfusion images. Background-suppressed pCASL images were scanned with a three-dimensional gradient- and spin-echo (GraSE) imaging readout module using a labeling pulse duration of 1.8 s and a post-labeling delay of 2 s. No flow-crushing gradients were applied. Other scan parameters were: field of view 160 × 160 mm; nominal voxel size, 2.0 × 2.0 × 6.0 mm^3^; 26 slices; repetition time/echo time, 4264/12 ms; flip angle, (refocusing pulses), 90°; and acquisition time, 4 min and 18 s. ASL-CBF maps were generated using the Basil tool of FSL, as described [10].

### 2.3. ASL Analysis

#### 2.3.1. Patients with Focal Epilepsy

All patients were investigated during the “interictal” phase, i.e., at least a minimum of 48 h after the last seizure. Two experts pediatric neuroradiologists with 25 and 12 years of experience visually inspected ASL CBF maps of patients with focal epilepsy in axial planes for identifying qualitative perfusion abnormalities. Readers performing this qualitative analysis were blinded to any hypotheses regarding electro-clinical SOZ. A suspected perfusion abnormality had to be seen on more than 2 consecutive slices to be considered positive.

CBF maps of patients were further analyzed to quantify the index of asymmetry (AI), which identifies voxels with significant differences in CBF between brain hemispheres [14]. Since all patients suffered from unilateral focal epilepsy, the brain regions with significant inter-hemispheric differences in CBF were considered presumed SOZ. The calculation of AI was based on the method described by Boscolo Galazzo et al. [14]. Briefly, CBF maps in the ASL space were affine-registered to the individual 3D-T1 high-resolution anatomical images by using the FLIRT tool of FSL. Each T1-weighted image was then registered to the MNI (Montreal Neurological Institute) space with 1 × 1 × 1 mm^3^ resolution using a non-linear method (FNIRT tool in FSL). Finally, the joint ASL/T1-weighted and T1-weighted/MNI space transformation parameters were combined to spatially normalize the CBF maps in the MNI space. The registered CBF maps were then smoothed with a 2 mm FWHM Gaussian kernel. Then, a voxel-wise AI calculation was performed using the following formula: AI = 100 × (Right − Left)/(Right + Left). After calculating the mean and standard deviation of the whole set of AI values, a voxel-wise AI z-score map was derived as: AI-zscore= [(AI value–mean AI)/standard-deviation AI]. Voxels with | AI-zscore | ≥ 1.64 corresponding to *p* < 0.05 were finally considered to have significantly different CBF between hemispheres and were therefore likely related to the presumed SOZ.

A further quantitative voxel-based analysis was performed to identify brain regions where CBF data deviated significantly from normative ASL data sets of age-matched healthy controls. In particular, we assumed that brain regions showing a significant discrepancy of CBF from healthy controls can be considered likely related to the SOZ. For this analysis, CBF maps in the ASL space were affine-registered to the individual 3D-T1 high-resolution anatomical images by using the FLIRT tool of FSL. Each T1-weighted image was then registered to the MNI space with 1 × 1 × 1 mm^3^ resolution using a non-linear method (FNIRT tool in FSL). Finally, the joint ASL/T1-weighted and T1-weighted/MNI space transformation parameters were combined to spatially normalize the CBF maps in the MNI space. CBF maps in the MNI space of each patient were first smoothed with a 2 mm FWHM Gaussian kernel and then processed to derive a z-score map of the difference from the age-matched CBF normative data set using the following formula: cCBF-zscore = [(CBF value − mean of normative CBF)/standard deviation of normative CBF]. We assumed a normal distribution of the CBF values; accordingly, voxels with |cCBF-zscore| ≤ −1.64 (corresponding to *p* < 0.05) were considered to have significantly lower CBF compared to the controls and, therefore, related to the presumed SOZ.

#### 2.3.2. Healthy Controls

CBF maps of healthy controls were first qualitatively evaluated in consensus by two experts, pediatric neuroradiologists, to define the presence of CBF asymmetry between brain hemispheres. Accordingly, they classified control subjects into two groups: (i) with a normal CBF map (no asymmetry), and (ii) with an abnormal CBF map (presence of asymmetry). To confirm this first qualitative evaluation, the CBF maps of patients classified into the normal group were further evaluated using a voxel-based quantitative approach that quantified the asymmetry index (AI). The same aforementioned method was used to calculate AI inpatients with epilepsy [14]. Only subjects without significant asymmetry of CBF between hemispheres were considered in the control group.

For each age group of controls, we calculated mean and standard deviation of CBF using the fsl maths function of FSL, and we finally considered these data as normative references for the quantitative analyses of cCBF.

#### 2.3.3. Definition of the Presumed Seizure Onset Zone

Given that the sample was etiologically dissimilar and that most of the subjects did not have surgery, we decided to consider the concept of the Seizure Onset Zone and to avoid the term Epileptogenic Zone [3], since our aim was not strictly related to the concept of surgery.

In cases of MR-positive epilepsy, the lesion identified on the MRI was considered as epileptogenic (macroscopic lesion that is causative of epileptic seizures because the lesion itself is epileptogenic or by secondary hyperexcitability of the adjacent cortex) [18], if consistent with electro-clinical SOZ. Moreover, confirmation of the SOZ location was obtained by a seizure-free outcome (1 year follow-up) after surgical resection of brain lesions in a restricted subgroup of patients undergoing surgical resection. For patients with negative MRI, presumed SOZ was determined by an expert consensus after reviewing clinical history, interictal and ictal scalp VEEG, structural MRI, interictal PET, and Electrical Source Imaging (when available). These localizations are referred to as presumed, since they were not supported by surgical outcomes.

#### 2.3.4. Concordance of ASL with SOZ

To compare ASL data (resulting from both qualitative and quantitative analyses) with the presumed electro-clinical SOZ, the following brain segments were classified and thus considered [15]:

a. Frontal lobe: orbitofrontal (inferior surface), mesial frontal (medial to the interhemispheric fissure), anterior lateral frontal (anterior to the precentral sulcus), posterior lateral frontal (anterior to the central sulcus, and posterior to the precentral sulcus) segments.
b. Temporal lobe: lateral anterior and posterior segments and temporal-mesial segment.
c. Parietal lobe: post-central, superior (superior to intraparietal sulcus), and inferior parietal (inferior to intraparietal sulcus) segments.
d. Occipital lobe (without subdivisions).

The brain segments, including the presumed SOZ defined by ASL qualitative and quantitative analyses, were compared with the presumed SOZ defined by anatomo-electro-clinical data and then classified in consensus as one of the following definitions by a neurologist and a neuroradiologist:

a. Concordant: If the SOZ defined by anatomo-electro-clinical data overlapped entirely with the one identified by the ASL.
b. Partially concordant: If the SOZ defined by anatomo-electro-clinically partially overlapped with the one identified by the ASL.
c. Discordant-ipsilateral: If the anatomo-electro-clinical SOZ and the one identified by the ASL were different regions of the same hemisphere.
d. Discordant-contralateral: If the anatomo-electro-clinical SOZ and the one identified by the ASL were in opposite hemispheres.
e. Uninformative: If no localization can be inferred.

### 2.4. Statistical Analysis

The data were analyzed using SPSS Statistics software, v26 (IBM, Armonk, NY, USA). Continuous variables were reported as mean and standard deviation, while categorical variables were expressed as absolute frequency and relative percentage. Pearson’s chi-squared test was used to compare the detection scores of the three ASL evaluations, and adjusted standardized residuals were evaluated for the post-hoc analysis [19]. Statistical significance was set at *p* < 0.05, and a Bonferroni correction was used to adjust for multiple comparisons.

The inter-rater agreement between three sets of ASL analyses and the anatomo-electro-clinical definition of SOZ was measured with Cohen’s κ coefficient. This statistic takes into effect the percentages of agreement that would be expected by chance. Possible values for the kappa statistic are from −1 to 1, with 1 = perfect agreement, 0 = completely random agreement and −1 = perfect disagreement. We interpret values between 0.0 and 0.2 to indicate slight agreement, 0.21 and 0.40 to indicate fair agreement, 0.41 and 0.60 to indicate moderate agreement, 0.61 and 0.80 to indicate substantial agreement, and 0.81 and 1.0 to indicate almost perfect agreement [20].

## 3. Results

### 3.1. Patients

Overall, 70 pediatric patients with unilateral focal epilepsy, studied with 3D-PCASL perfusion were considered in this study. Five were excluded for motion artifacts. Thus, 65 pediatric patients with electro-clinically defined focal epilepsy were finally considered for the analysis. Twenty-six patients (11 female; mean age 11.5 ± 3.6 years) presented with lesional focal epilepsy (18 focal cortical dysplasia, 4 glioneuronal tumors, 1 transmantle gray matter heterotopia, 2 cavernous venous malformations, 1 single cortical tuber). Fifteen of them underwent surgical resection of the brain lesion. Fourteen out of the 15 operated patients were seizure-free (Engel Class I), although only 12 had a 1-year follow-up, with the remaining patient in Engel class III. Among non-operated MRI-positive patients (11 pts), surgery was scheduled for two, an in-depth study with SEEG was programmed for other three, in a sixth patient surgical planning was stopped due to the overlapping of EZ and an eloquent area, and three other patients were drug-responsive; thus, a “watchful waiting” approach was agreed with the families. Finally, surgery was proposed to two other patients’ families, who were lost in follow-up. The remaining 39 patients (17 female; mean age 10.6 ± 4.7 years) showed MRI-negative unilateral focal epilepsy, including patients with presumed structural etiology not detectable on structural MRI and patients with non-lesional epilepsy [21].

A total of 100 subjects with normal brain MRI and without CBF asymmetry at perfusion analyses were included in the group of healthy controls. Specifically, 50 of them were younger than 7 years old (22 female; mean age 5.2 ± 2.3 years), and the remaining 50 subjects were older or equal to 7 years old (27 female; mean age 10.6 ± 4.1 years).

### 3.2. ASL Analyses

Table 1 summarizes the scores of the three ASL analyses for the identification of ASL abnormality in comparison with the presumed anatomo-electro-clinical SOZ and electro-clinical SOZ in MRI-positive and MRI-negative patients, respectively.

### 3.3. Qualitative Analysis

In the group of positive-MRI patients, 7 out of 26 SOZ (27%) were correctly identified, with good agreement (Cohen’s k = 0.732) for the definition of lateralization. The location of the CBF abnormality was concordant with the anatomo-electro-clinical SOZ for 5/26 patients, and partially concordant for 2/26 patients. In the remaining 19 positive-MRI patients, the localization was discordant (1 ipsilateral, and 4 contralateral), or uninformative (14 cases).

In negative-MRI patients, 7 out of 39 presumed SOZ (18%) were correctly identified. Definition of ASL abnormality lateralization showed fair agreement with electro-clinical presumed SOZ (Cohen’s k = 0.381). The location of the CBF abnormality was concordant with the electro-clinical presumed SOZ for 4/39 patients, and partially concordant for 3/39 patients. In the remaining 32 negative-MRI patients, the localization was discordant ipsilateral (1 case) or uninformative (3 cases).

### 3.4. Asymmetry Index

In the group of positive-MRI patients, 19 out of 26 SOZ (73%) were correctly identified, with almost perfect agreement (Cohen’s k = 0.909) for the definition of lateralization. The location of the CBF abnormality was concordant with the anatomo-electro-clinical presumed SOZ for 14/26 patients, and partially concordant for 5/26 patients. In the remaining 7 positive-MRI patients, the localization was discordant (3 ipsilateral, and 1 contralateral), or uninformative (3 cases).

In negative-MRI patients, 18 out of 39 presumed SOZ (46%) were correctly identified. Definition of ASL abnormality lateralization showed good agreement with electro-clinical presumed SOZ (Cohen’s k = 0.819). The location of the CBF abnormality was concordant with the electro-clinical presumed SOZ for 6/39 patients, and partially concordant for 12/39 patients. In the remaining 21 negative-MRI patients, the localization was discordant (5 ipsilateral and 1 contralateral) or uninformative (15 cases).

### 3.5. ASL cCBF Analysis

In the group of positive-MRI patients, 20 out of 26 SOZ (77%) were correctly identified, with almost perfect agreement (Cohen’s k = 0.943) for the definition of lateralization. The location of the CBF abnormality was concordant with the anatomo-electro-clinical presumed SOZ for 14/26 patients, and partially concordant for 6/26 patients. In the remaining 6 positive-MRI patients, the localization was discordant (4 ipsilateral) or uninformative (2 cases).

In negative-MRI patients 25 out of 39 presumed SOZ (64%) were correctly identified. The definition of ASL abnormality lateralization showed almost perfect agreement with electro-clinical presumed SOZ (Cohen’s k = 0.932). The location of the CBF abnormality was concordant with electro-clinical presumed SOZ for 11/39 patients, and partially concordant for 14/39 patients. In the remaining 14 negative-MRI patients, the localization was discordant (5 ipsilateral and 2 contralateral) or uninformative (7 cases).

### 3.6. Agreement among ASL Analyses

In the group patients with a positive MRI, only 6/26 (23%) SOZs, concordant or partially concordant with anatomo-electro-clinical localization, were correctly identified by all three ASL analyses. Similarly, in the group of negative MRI patients, 6/39 (15%) SOZs concordant or partially concordant with electro-clinical localization were correctly identified by all three ASL analyses.

**Figure 1:**
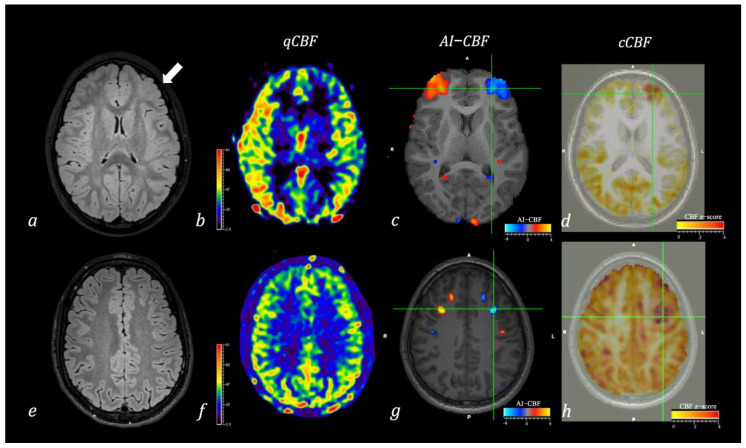
Two cases of pediatric patients with focal epilepsy and frontal left EEG abnormalities. In the first patient (**a**–**d**), axial FLAIR image (**a**) shows left frontal focal cortical dysplasia (white arrow), while axial ASL-CBF map (**b**) identified a slight reduction of CBF in the left frontal region. Quantitative voxel-based analysis of the Asymmetry Index (**c**) confirmed a region of asymmetry of CBF in the frontal lobes with reduced perfusion in the left side. Quantitative voxel-base analysis of cCBF (**d**) identified a region of significant reduction of CBF in the left frontal lobe compared with age-matched healthy controls. In the second patient (**e**–**h**), axial FLAIR image (**e**) and axial ASL-CBF map (**f**) did not show any structural lesion or perfusion abnormalities at visual qualitative analysis. In contrast, quantitative voxel-based analysis of the Asymmetry Index (**g**) showed a region of asymmetry of CBF in frontal lobes with reduced perfusion in the left side. Quantitative voxel-base analysis of cCBF (**h**) identified a region of significant reduction of CBF in the left frontal lobe compared with age-matched healthy controls. Note: Color bars in (**b**,**f**) indicate CBF values mL/min/100 g. Color bars in (**c**,**g**) indicate values of the Asymmetry index with red-to-yellow indicating positive values and blue-to-lightblue indicating negative values. Colorbars in (**d**,**h**) indicate z-score values of the CBF comparison between the single subject and healthy controls.

Focusing on the quantitative ASL analyses, 16/26 (61%) and 16/39 (41%) SOZs concordant or partially concordant with their anatomo-electro-clinical localizations were correctly localized by both AI-CBF and cCBF analyses in the group of positive- (X^2^ = 38.035; *p* < 0.001) and negative-MRI patients (X^2^ = 26,761; *p* = 0.044), respectively (Figure 1). Of note, one patient in the positive-MRI group and five patients in the negative-MRI group who did not show concordant results in the AI-CBF analysis were found to be concordant in the cCBF analysis. In contrast, no patients who did not show concordant results in the cCBF analysis were found to be concordant in the AI-CBF analysis.

The detection scores of both AI-CBF and cCBF were significantly higher than qCBF in both MRI-positive (*p* ≤ 0.001) and MRI-negative (*p* ≤ 0.007) patients at post-hoc analysis. No significant differences were observed between cCBF and AI-CBF (Table 2).

**Figure 2:**
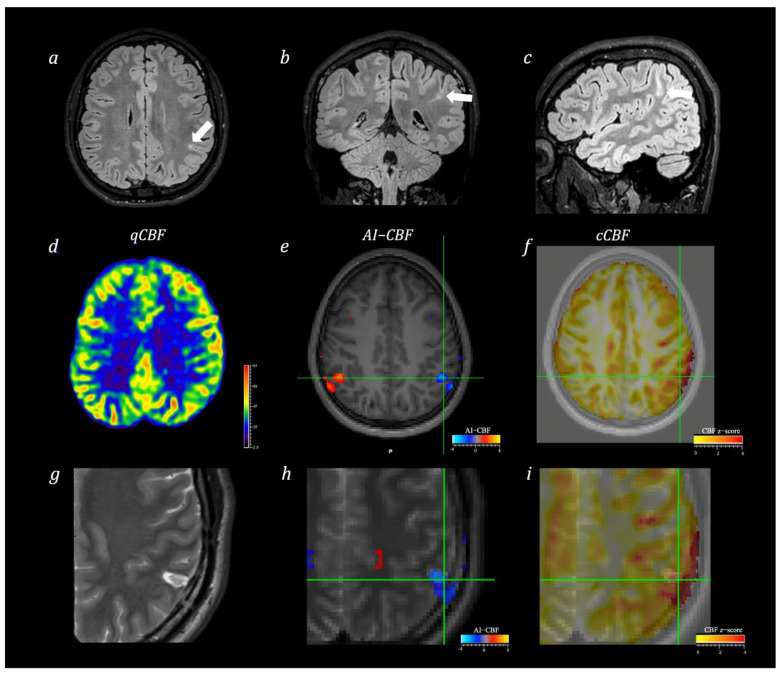
MR images of Engel class I patient with focal left parietal epilepsy undergoing surgery. 3D-FLAIR images (**a**–**c**) show left parietal bottom-of-sulcus cortical dysplasia (white arrows). Qualitative analysis of the ASL-CBF map (**d**) identified a slight reduction of CBF in the left parietal region. Quantitative voxel-based analysis of the CBF Asymmetry Index (**e**) confirmed a region of asymmetry in the parietal lobes with reduced perfusion in the left side. Quantitative voxel-base analysis of cCBF (**f**) identified in the same region of the left parietal lobe a region of significant reduction of CBF compared with age-matched healthy controls. Axial T2-weighted image acquired after surgery (**g**) shows a resected area in the left parietal lobe. AI-CBF (**h**) and cCBF z-score maps (**i**) overlaid on post-surgery T2-weighted image show perfect correspondence of the cluster of significant results with the area of resected lesion.

Similar results were obtained comparing the three ASL analyses in the subgroup of patients with positive MRI undergoing surgical resection (15 out of 26 patients) (X^2^ = 17.253; *p* < 0.001). In particular, the detection score of operated lesions was significantly higher for both AI-CBF and cCBF analyses compared to qASL (*p* = 0.02 and *p* = 0.01, respectively) (Table 3). No significant differences were observed between AI-CBF and cCBF (*p* = 0.511) (Figure 2). 

## 4. Discussion

In this study, we found that quantitative voxel-based ASL analyses improve the detection rate of SOZ in pediatric patients with focal epilepsy with both positive and negative brain MRI studies, compared with the qualitative visual analysis of CBF maps. Our results suggest ASL as a safe and manageable imaging technique that can be adopted to assist in the localization of SOZ in pediatric focal epilepsy in clinical practice.

Indeed, ASL MR perfusion employs magnetically labeled blood water protons as endogenous diffusible tracers to noninvasively estimate whole-brain perfusion. Moreover, ASL provides an absolute quantification of CBF in different brain regions that do not require exogenous contrast injection or exposure to ionizing radiation, which is suitable for patients who require multiple MR evaluations. Several studies have already analyzed the localization value of ASL in patients with epilepsy, such as in MRI-negative children with new onset seizures, mesial temporal lobe epilepsy, intractable epilepsy, partial epilepsy status, and tuberous sclerosis [22,23,24,25,26]. Epilepsy generally induces cortical hyperperfusion during seizures and in the peri-ictal period (≤5 h from the last seizure), which is likely related to the extreme electrophysiological state, with the activated cortex exhibiting increased glucose and oxygen usage leading to increased local CBF in the SOZ [27,28]. The origin of relative hypoperfusion occurring during the interictal phase is not fully understood. This could indicate local brain atrophy and gliosis. Indeed, with the prolonged course of the disease, a progressive reduction of local neurons can be observed within the SOZ, thus resulting in reduced CBF during the interictal phases [23]. On the other hand, the focal hypometabolism during the interictal state of the SOZ might be related to its functional and metabolic isolation from the surrounding brain regions [29,30].

Although several visual qualitative control score systems have been developed to provide standardized quality indications before using ASL for clinical assessments [31], the ability of ASL to identify the SOZ is still unsatisfactory when perfusion maps are assessed with a qualitative visual approach. Of note, So Me Lee et al. analyzed perfusion abnormalities on ASL in 43 pediatric patients with newly developed seizures who did not show abnormalities in structural MRI using a qualitative visual approach, finding perfusion change in only 58.1% of the patients, with moderate concordance (k = 0.542) with the electro-clinical SOZ based on semiology and EEG findings [25]. Better results have been reported in additional case series that analyzed the diagnostic utility of ASL on seizure evaluation in pediatric epilepsy with focal structural brain abnormalities (i.e., focal cortical dysplasia [32], cortical tubers [26], and focal brain lesions in neonatal periods [33]). In our study, we obtained similar results from the qualitative analysis of ASL maps that allowed us to identify 27% and 18% of electro-clinical SOZs in MRI positive and negative patients’ groups, respectively, confirming the low accuracy of the qualitative analyses of ASL in the localization of SOZ in the clinical setting.

Moreover, we obtained better results from both quantitative voxel-based analyses of CBF maps. In particular, we found that cCBF analysis properly localizes the highest number of SOZs concordant with the electro-clinical findings (77% and 64% of cases in MRI-positive and MRI-negative patients). These results are in agreement with previous studies performed on adult patients with focal epilepsy. In particular, Pereira et al. found that statistical maps obtained comparing single-subject CBF maps with healthy controls may provide localizing or lateralizing information for specific cases that were missed through qualitative analysis of ASL maps [15]. This higher sensitivity of cCBF analysis may be related to its ability to reveal minimal variations of CBF from normal values, which might otherwise not be identified through visual qualitative assessments of ASL maps. However, these improvements in ASL sensitivity in the identification of SOZ were observed in the face of a lower specificity. Indeed, we observed the highest numbers of cases with “partially concordance” between ASL and electro-clinical data (6 out of 26 MRI-positive cases and 14 out of 39 MRI-negative cases) in the identification of SOZ using the quantitative cCBF approach. This might reflect the greater number of cases with multifocal significant perfusion changes that were seen with the cCBF analysis, where some areas of significant hypoperfusion were outside the SOZ defined by the electro-clinical analysis.

Interestingly, there is increasing evidence to consider focal epilepsy as a brain network disease in which long-range connections need to be taken into account [34]. Of note, the presence of perfusion alterations not only in the regions of seizure onset might support the presence of complex altered epileptic networks with perfusion abnormalities involving seizure propagation pathways [35]. These findings suggest that quantitative cCBF analysis may be useful for a better localization of single seizure foci, as well as for understanding the widespread epileptic networks involved by the EZ.

Moreover, our analysis revealed a higher specificity of AI-CBF analysis in the localization of SOZ. Indeed, AI-CBF revealed fewer “Partially concordant” and “Discordant ipsilateral” cases than cCBF. The analysis of AI-CBF is mainly based on the voxel-wise calculation of perfusion asymmetries between the two hemispheres. This approach has already been successfully applied in previous studies and proved clinically useful for detecting focal abnormalities in both perfusion and metabolic maps of patients with epilepsy [12,36,37]. Of note, voxel-wise AI-CBF data were evaluated in a two-fold way: (i) comparing the estimated AI values to a reference threshold derived from a control group to identify the significant voxels of asymmetry [38,39] or (ii) using a qualitative analysis of asymmetry in the brain regions of the presumed SOZ [12]. In this study, we used a novel approach of analysis of AI maps, which applies an individually-tailored z-score test to the estimated AI values using the information derived from each patient, to automatically identify areas with statistically significant asymmetries [14]. This approach allows us to perform a quantitative voxel-based analysis of a single patient without using the information derived from a normal database, thus overcoming the limitations imposed by the lack of normative data. In particular, we used AI-CBF z-values with thresholding |zAI-CBF| >  1.64 (corresponding to *p* < 0.05), and we considered as presumed SOZ all clusters of voxels above this threshold. Interestingly, several new methods for subject-specific adaptive thresholding were recently tested to improve the sensitivity and specificity of AI-CBF to localize SOZ [40]. More specifically, the computation of a preliminary subject-specific AI-CBF histogram allows us to derive different thresholds (i.e., minimal product criterion, minimal distance criterion, and elbow criterion) that showed better results in terms of positive predictive and true positive rate of SOZ localization [40]. However, these new methods require additional steps and specific tools for AI-CBF analysis that may represent a limitation for clinical use.

Furthermore, the identification of techniques to locate lesions before surgery has become the key to presurgical evaluation. Structural brain MRI and Video EEG are effective investigation methods for EZ localization. However, in the presurgical evaluation of pediatric epilepsy, the occurrence of negative findings in MRI can be as high as 30–40% [15]. Therefore, it is essential to find alternative approaches to improving the sensitivity of MRI in patients with refractory focal epilepsy for presurgical evaluation.

Although many patients with epilepsy have visible structural lesions, the most challenging group is the subset of patients that have no structural or functional abnormalities visible on MRI, and the question remains how to improve localization of EZ in this group. We found that ASL was able to identify regions of perfusion change in 18 out of 39 (46%) patients with AI-CBF analysis (6 “Concordant” and 12 “Partially concordant” with electro-clinical SOZ), and in 25 out of 39 patients (64%) with cCBF analysis (11“Concordant” and 14 “Partially concordant” with electro-clinical SOZ). On the contrary, the qualitative analysis detected only perfusion changes in 7 out of 39 patients (18%) without structural brain lesions on MRI, thus confirming the low diagnostic performance of the visual approach reported in previous studies performed on adults. In particular, Lam et al. [41] and Sierra-Marcos et al. [42] reported a very poor detection rate in their non-lesional group using a visual analysis of ASL maps. In contrast, a more recent study in adults evaluating AI-CBF reported ASL hypoperfusion, which was concordant with the final electro-clinical hypothesis in 13 of 20 patients with MRI-negative [14]. In our study, we showed that both ASL quantitative analyses may improve the detection of SOZ in children with uninformative MRI, and we suggest using both methods in more complex clinical cases with subtle MRI signal abnormalities that are not directly obvious from the radiological interpretation of MRI. This may substantially help in refining the final decision of the SOZ location in surgery candidates.

It is worth noting that this should be considered a hypothesis, since, in order to prove the added value of the ASL in presurgical assessment, the concordance/discordance of zones identified by different methods should be analyzed according to postoperative outcome (i.e., Engel class) and the accuracy of planned cortical resection.

In this regard, the majority of subjects in the subgroup undergoing epilepsy surgery (92% Engel class I) presented good concordance between the results of quantitative ASL analyses and anatomo-electro-clinical SOZ. On the other hand, poor concordance was observed in the qASL analysis. These findings should also be confirmed in patients with MRI negative patients submitted to surgery after SEEG.

There are several limitations of our study. In particular, the localization accuracy of ASL depends on the quality of the sequence in terms of noise and motion artifacts and signal-to-noise ratio. For example, there could be substantial noise artifacts close to bone and scalp on ASL maps, which may affect visualization and CBF calculation in adjacent cortical areas. In addition, differences in perfusion of white matter are difficult to detect due to the inherent low perfusion of white matter. However, the aim of ASL is to localize perfusion changes within the cerebral cortex.

Moreover, all patients were studied during the inter-ictal period (i.e., at least 48 h from the last seizure), thus not thoroughly considering the effect on cerebral perfusion of time elapsed since the last seizure, with special regard to the peri-ictal period, i.e., within 48 h of seizure.

Furthermore, we did not evaluate the influence of antiseizure medications on the ability of ASL to localize SOZ [43]. Further studies might provide further insights into these aspects.

In conclusion, our findings suggest that advanced quantitative voxel-based analyses, performed on ASL data could provide additional information for the localization of the SOZ in pediatric patients with focal epilepsy. While waiting for further studies to validate the added value in the epilepsy surgery work-up, due to the relative convenience and noninvasive nature, we suggest that ASL be performed as part of the presurgical evaluation in all children with focal epilepsy, as well as in those with a negative MRI. Indeed, the added value of quantitative ASL analyses could be for patients with focal epilepsy in whom no lesions or controversial signal abnormalities are seen on an MRI. In these cases, advanced quantitative analyses of ASL data might be integrated in a multimodal evaluation in combination with other advanced methods available for the presurgical protocol (i.e., DTI, PET, and SPECT, Electrical Source Imaging) [44].

## Figures and Tables

**Table 1:** Comparisons of three ASL analyses with the electro-clinical definition of the presumed Seizure Onset Zone (SOZ).

	qASL			AI-CBF			cCBF		
	SOZ *(%)*	SOZ Lateralization Agreement *Cohen’s Kappa* *(95% CI)*	Brain Lobe Agreement	SOZ *(%)*	SOZ Lateralization Agreement *Cohen’s Kappa* *(95% CI)*	Brain Lobe Agreement	SOZ *(%)*	SOZ Lateralization Agreement *Cohen’s Kappa* *(95% CI)*	Brain Lobe Agreement
MRI positive 26 patients	7/26 *(27%)*	0.392 (0.211–0.398)	Concordant: 5/26 Partially concordant: 2/26 Discordant ipsilateral: 1/26 Discordant contralateral: 4/26 Uninformative: 14/26	19/26 *(73%)*	0.909 (0.891–0.927)	Concordant: 14/26 Partially concordant: 5/26 Discordant ipsilateral: 3/26 Discordant contralateral: 1/26 Uninformative: 3/26	20/26 *(77%)*	0.943 (0.885–0.968)	Concordant: 14/26 Partially concordant: 6/26 Discordant ipsilateral: 4/26 Discordant contralateral: 0/26 Uninformative: 2/26
MRI negative 39 patients	7/39 *(18%)*	0.385 (0.160–0.336)	Concordant: 4/*39* Partially concordant: 3/39 Discordant ipsilateral: 1/39 Discordant contralateral: 0/39 Uninformative: 31/39	18/39 *(46%)*	0.819 (0.796–0.842)	Concordant: 6/39 Partially concordant: 12/39 Discordant ipsilateral: 5/39 Discordant contralateral: 1/39 Uninformative: 15/39	25/39 *(64%)*	0.932 (0.896–0.941)	Concordant: 11/39 Partially concordant: 14/39 Discordant ipsilateral: 5/39 Discordant contralateral: 2/39 Uninformative: 7/39

**Table 2:** Comparison of ASL analyses.

ASL Analyses		MRI-Positive 29 Patients	MRI-Negative 36 Patients
Method 1	Method 2	*p*-Value *	*p*-Value *
qCBF	AI-CBF	**0.001**	**0.007**
qCBF	cCBF	**<0.001**	**<0.001**
AI-CBF	cCBF	0.500	0.086

Note: qCBF indicates qualitative analysis of ASL images; AI-CBF indicates quantitative analysis of Asymmetry index; cCBF indicates quantitative analysis of CBF of each patient with baseline normative ASL data. * The *p*-value indicates the results of post-hoc analyses of Chi-square test.

**Table 3:** Comparisons of three ASL analyses in the subgroup of patients with a positive MRI undergoing surgery. Concordance was evaluated between the site of the resected lesion and the area identified as SOZ at ASL analyses.

ASL Analyses	Concordance between Site of Lesion and ASL Results	*p*-Values
qASL	Concordant: 5/15 Partially concordant: 2/15 Discordant ipsilateral: 4/15 Discordant contralateral: 1/15 Uninformative: 3/15	qASL vs AI-CBF: **0.02** qASL vs cCBF: **0.01**
AI-CBF	Concordant: 11/15 Partially concordant: 2/15 Discordant ipsilateral: 1/15 Discordant contralateral: 0/15 Uninformative: 1/15	AI-CBF vs qASL: **0.02** AI-CBF vs cCBF: 0.511
cCBF	Concordant: 11/15 Partially concordant: 3/15 Discordant ipsilateral: 1/15 Discordant contralateral: 0/15 Uninformative: 0/15	cCBF vs qASL: **0.01** cCBF vs AI-CBF: **0.511**

Note *p*-values indicate statistical levels in post-hoc analysis based on the analysis of adjusted standardized residuals.

## Data Availability

Data used for the analysis can be requested by correspondence with domenicotortora@gaslini.org.
